# DNA methylation mediates the effect of maternal cognitive appraisal of a disaster in pregnancy on the child’s C-peptide secretion in adolescence: Project Ice Storm

**DOI:** 10.1371/journal.pone.0192199

**Published:** 2018-02-05

**Authors:** Lei Cao-Lei, Kelsey N. Dancause, Guillaume Elgbeili, David P. Laplante, Moshe Szyf, Suzanne King

**Affiliations:** 1 Department of Psychiatry, McGill University and Douglas Hospital Research Centre, Montreal, Quebec, Canada; 2 Department of Physical Activity Sciences, University of Quebec at Montreal, Montreal, Quebec, Canada; 3 Douglas Hospital Research Centre, Montreal, Quebec, Canada; 4 Department of Pharmacology and Therapeutics and Sackler Program for Epigenetics and Developmental Psychobiology, McGill University, Montreal, Quebec, Canada; Inc, UNITED STATES

## Abstract

Animal and human studies suggest that prenatal exposure to stress is associated with adverse health outcomes such as type 2 diabetes. Epigenetic modification, such as DNA methylation, is considered one possible underlying mechanism. The 1998 Quebec ice storm provides a unique opportunity to study an independent prenatal stressor on child outcomes. C-peptide is the best measure of endogenous insulin secretion and is widely used in the clinical management of patients with diabetes. The objectives of this study are to determine 1) the extent to which prenatal exposure to disaster-related stress (maternal objective hardship and maternal cognitive appraisal) influences children’s C-peptide secretion, and 2) whether DNA methylation of diabetes-related genes mediates the effects of prenatal stress on C-peptide secretion. Children’s (n = 30) C-peptide secretion in response to an oral glucose tolerance test were assessed in blood at 13½ years. DNA methylation levels of selected type 1 and 2 diabetes-related genes were chosen based upon the genes associated with prenatal maternal objective hardship and/or cognitive appraisal levels. Bootstrapping analyses were performed to determine the mediation effect of DNA methylation. We found that children whose mothers experienced higher objective hardship exhibited higher C-peptide secretion. Cognitive appraisal was not directly associated with C-peptide secretion. DNA methylation of diabetes-related genes had a positive mediation effect of objective hardship on C-peptide secretion: higher objective hardship predicted higher C-peptide secretion through DNA methylation. Negative mediation effects of cognitive appraisal were observed: negative cognitive appraisal predicted higher C-peptide secretion through DNA methylation. However, only one gene, *LTA*, remained a significant mediator of cognitive appraisal on C-peptide secretion after the conservative Bonferroni multiple corrections. Our findings suggest that DNA methylation could act as an intervening variable between prenatal stress and metabolic outcomes, highlighting the importance of epigenetic mechanisms in response to environmental factors.

## Introduction

The prevalence of metabolic diseases has recently escalated among children and adolescents, holding lifelong implications for health [1]. Animal studies suggest that prenatal exposure to stress or glucocorticoids (GC) is associated with type 2 diabetes, hyperglycemia, and features of insulin resistance [2–6]. Human studies similarly demonstrate that prenatal exposure to high levels of GCs, either exogenous or as a consequence of prenatal maternal stress (PNMS), is associated with poor fetal growth [7–9] and the deregulation of the HPA axis [10], which is involved in metabolic pathways [11] and likely represents one pathway linking PNMS with later metabolic outcomes [12–15]. Furthermore, higher levels of PNMS exposure are associated with low birth weight and intrauterine growth restriction [12, 15] which are both associated with increased metabolic disease risk [16, 17].

The impact of PNMS on specific features of glucose-insulin metabolism among humans is not entirely clear. Retrospective case-control studies [15, 18] suggest that there is increased risk of insulin resistance among young adults whose mothers experienced stressful life events during pregnancy: a PNMS group of young adults who reported that their mothers had had stressful life events during pregnancy showed elevated fasting insulin, elevated insulin after an oral glucose tolerance test, higher C-peptide levels, and decreased low density lipoprotein (LDL) levels compared to a non-stressed comparison group, even after controlling for body mass index (BMI) and other potential confounding variables [18]. However, the conclusions that one can draw from these data are limited by challenges that commonly affect human PNMS studies. First, retrospective approaches are unable to distinguish which aspect of the maternal stress experience is associated with the outcome. Second, depression or relationship difficulties during pregnancy are not randomly assigned; studies showing associations between maternal psychosocial characteristics or life events and child outcomes cannot disentangle the genetic transmission of parental traits from effects of the intrauterine environment and from conditions in the postnatal environment. Finally, mothers in retrospective studies may be biased in their reporting of life events based on their impressions of their children’s health outcomes. These limitations could be partly overcome by a human model analyzing a stressor that is independent of the parents’ potential influence, and whose severity is randomly distributed.

Natural disasters are such events. In January 1998, a series of ice storms caused power outages for more than 1.4 million Québec households during the coldest period of the year for periods ranging from a few hours to more than six weeks. The ice storm has been described as Canada’s most costly natural disaster in history [19, 20]. Project Ice Storm has been following a cohort of children whose mothers were pregnant during the Quebec ice storm. In June 1998 we assessed maternal stress levels in terms of their objective degree of hardship experienced, the mothers’ subjective distress, and their cognitive appraisal of the disaster. Analyses from this cohort have shown that storm-related objective hardship and subjective distress are associated with cognitive [21, 22], linguistic [23, 24], immunological [25, 26], motor [27] and behavioral outcomes [28]. Furthermore, the severity of maternal objective stress exposure was associated with risk for obesity at 5½ years [29], with the PNMS effect increasing in magnitude between the ages of 5½ and 15½ [30].

Thus, results from Project Ice Storm demonstrate that an independent stressor can have long-term effects on later cardiometabolic outcomes, consistent with our expectations from animal studies and from retrospective studies of PNMS among humans. However, the underlying molecular mechanisms responsible for these effects are still not well understood. Epigenetic processes, such as DNA methylation, are proposed to be strong candidates that mediate the effects of PNMS on metabolic health in offspring, but few studies are able to conduct empirical tests of these effects. Previous analyses of the Project Ice Storm cohort demonstrated that an epigenetic signature with alteration of the genome-wide DNA methylation pattern is triggered by both objective hardship exposure and maternal cognitive appraisal, but not by maternal subjective distress [31, 32]. Furthermore, we demonstrated a mediating role of DNA methylation of selected genes related to type 1 and 2 diabetes mellitus in the association between objective hardship and children’s body mass index (BMI) and measures of central adiposity [33]. Likewise, we reported the mediation effect of DNA methylation of genes related to NF-κB signaling pathway on the association between objective hardship and cytokine levels in 13½ year-old adolescents[34]. More recently, we reported the mediation effect of DNA methylation levels of genes related to type 1 and 2 diabetes mellitus on the relationship between prenatal maternal cognitive appraisal and adolescent BMI and central adiposity [35].

Previously, we reported that the objective hardship was significantly positively correlated with children’s insulin secretion as well as with BMI at age 13½, suggesting that prenatal stress independently predicts metabolic outcomes in adolescence [36]. C-peptide, which is formed during cleavage of insulin from proinsulin, is produced in equal amounts to insulin and is not removed by the liver. Therefore, C-peptide is the best measure of endogenous insulin secretion and is widely used in the clinical management of patients with diabetes [37]. Thus, the objectives of this study were to determine 1) the extent to which prenatal exposure to disaster-related stress (maternal objective hardship and maternal cognitive appraisal) influences children’s C-peptide secretion, and 2) whether DNA methylation of diabetes-related genes mediates the effects of prenatal stress on C-peptide secretion. As no DNA methylation level was found to be associated with subjective distress in our previous analyses [31, 32], we excluded this predictor variable from our mediation analyses.

## Materials and methods

### Participants

In June 1998, 5 months after the onset of the ice storm, we identified women who were 18 years or older, and who were pregnant on January 9, 1998 (the peak of the ice storm), or who became pregnant within 3 months of that date. Participants were a subset of our Project Ice Storm cohort who had been assessed multiple times since birth. Blood was obtained from 32 13½ year-old adolescents; however, data from two adolescents were excluded as their DNA methylation data were missing. Therefore, data from 30 adolescents (18 boys and 12 girls) were used for the analyses. All participants were French-speaking Caucasians.

All phases of this study were approved by the Research Ethics Board of the McGill University-affiliated Douglas Hospital Research Center in Montreal, Canada. We obtained written informed consent from parents and written assent from children.

### Child metabolic outcome measures at age 13½ years

The blood sample collection has been described in greater detail elsewhere [38]. Briefly, we collected venous blood samples after an overnight fast, followed by collection 30 minutes after an oral glucose challenge (1.75g/kg, maximum 75g). Serum C-peptide levels were analyzed using chemiluminescent immunoassay at Ste. Justine Hospital, Montreal. Fasting and stimulated C-peptide levels were used for the current study. C-peptide secretion was estimated using the C-peptide-genic index [(C30–C0)/(G30–G0)]. The raw data are shown in S1 Table.

### Child birth outcome measures

Children’s birth weight, birth length, birth ponderal index and gestational age were obtained from maternal reports (transcribed from Quebec birth records given at discharge) 6 months after the birth, and from hospital records.

### Predictor variables (The raw data are shown in S1 Table)

#### Storm-related variables

At recruitment in June 1998, objective hardship was estimated using the mothers’ responses to questionnaire items tapping into categories of exposure used in other disaster studies: Threat, Loss, Scope, and Change [39]. Because each natural disaster presents unique experiences to the exposed population, questions pertaining to each of the four categories must be tailor-made. Each of the four dimensions was scored on a scale of 0–8, ranging from low exposure to high exposure. A total objective hardship score (Storm32) was computed by summing scores from all four dimensions using McFarlane's approach [40]. A full presentation of the Storm32 items and scoring has been published previously [25].

The assessment of the mothers’ cognitive appraisal of the ice storm was also included in the recruitment questionnaire [32]. Briefly, we included the following item: “Overall, what were the consequences of the ice storm on you and your family?”; response options were on a five-point scale of “Very negative” (1), “Negative” (2), “Neutral” (3), Positive (4), and “Very positive” (5). In the subgroup of 30 mothers whose children participated in the blood studies, none had rated the consequences of the storm as “Very negative”, while 9 (30.0%) had given a rating of “Negative”, 4 (13.3%) had given a “Neutral” rating, 16 (53.3%) had given a rating of “Positive”, and 1 (3.3%) had considered the consequences “Very positive”. As our interest is the effect of negative cognitive appraisal about the ice storm on child outcomes, we compared the participants who had rated the consequences as negative (“Negative cognitive appraisal group, recoded as 0) with a “Positive cognitive appraisal group” (recoded as 1) combining the participants who had rated the consequences as neutral, positive or very positive.

#### Maternal variables

Maternal psychological functioning was assessed with a validated French version of the widely used General Health Questionnaire-28 (GHQ) [41]. The GHQ is a self-report screening tool for psychiatric symptoms and includes seven items in each of the anxiety, dysfunction, somatization, and depression sub-scales. Items are scored on a 4-point Likert scale indicating the degree to which each symptom was experienced in the preceding 2 weeks. In the present study, each item was re-scored as either 0 (a rating of 0 or 1) or 1 (a rating of 2 or 3), according to the Goldberg method [41], resulting in a minimum possible score of 0 and a maximum possible score of 28. The total score was used in analyses. The GHQ was included in the June 1998 recruitment questionnaire, and also when their children were 13½ years of age.

Exposure to other potentially stressful maternal life events was assessed in a questionnaire sent 6 months after each woman’s due date, and again at the 13½ year assessment. Women answered the Life Experiences Survey [42], a self-report measure that lists 57 life changes, such as death of a spouse or a work promotion. To keep the questionnaire length reasonable, we reduced this to 29 events by eliminating items not likely to have occurred in this sample (e.g., combat experience). At the 6-month postnatal questionnaire, women indicated events that occurred during the 6 months since the baby’s due date, the 9 months of pregnancy, and the 3 months before conception. At 13½ years, they reported on events occurring since the child’s 12^th^ birthday. Women gave the approximate date of each event experienced, and rated the impact of each event on a 7-point Likert scale ranging from “extremely negative” to “extremely positive.” The numbers of life events at each assessment were used in analyses.

Data on maternal and paternal age and education, and parental job classification, were collected in June 1998 and were used to compute a socio-economic status (SES) score as using Hollingshead Index criteria [43].

### Blood samples and T-cell isolation at 13½ years

Blood was collected from the subjects for T cell isolation and DNA extraction using methods which have been described previously [31]. Briefly, we isolated T cells from peripheral blood mononuclear cells (PBMCs) by immunomagnetic separation with Dynabeads CD3 (Dynal, Invitrogen). We performed DNA extraction from T cells using Wizard Genomic DNA Purification kit (Promega) according to the manufacturer’s instructions.

### Infinium Human Methylation 450 BeadChip Array and data analysis

Infinium Human Methylation 450 BeadChip Array and data analysis has been described previously [33]. Briefly, Infinium Human Methylation 450 BeadChip was used to determine DNA methylation levels in T cells from the 34 adolescents who originally participated the epigenetic study [31]. 11648 probes on chromosomes X and Y were excluded. CpGs with an inter-quartile range (IQR) less than 0.10 (i.e., 10% methylation difference) were not analyzed. To avoid artifacts due to hybridization bias, probes containing SNP with a minor allele frequency (MAF) ≥ 5% in the HapMap CEU population were removed. The remaining 10,553 probes were tested for association with the objective PNMS and cognitive appraisal scores. The Benjamini-Hochberg algorithm was used to correct for multiple testing by computing the false discovery rate (FDR) which was set at <0.2. All samples were analyzed at the same time on the same plate. Illumina 450K Methylation BeadChip analyses were completed using standard procedures and described previously [31]. Methylation data are publicly available in the Gene Expression Omnibus (GEO) database (accession number GSE72354).

### Selection of candidate genes for testing mediation by methylation

In previous studies with this sample, we identified genes from isolated T cells whose methylation signatures were associated with either maternal objective hardship levels [29] or cognitive appraisal of the ice storm [30]. In order to investigate the mediating effect of DNA methylation on insulin and C-peptide at age 13½ years, we matched genes whose methylation had been significantly correlated with objective hardship or cognitive appraisal to the type 1 and 2 diabetes mellitus pathways as classified by IPA software (www.ingenuity.com). In total, 19 genes (64 CpGs) and 12 genes (19 CpGs) associated with objective hardship were selected from the type 1 and 2 diabetes mellitus pathway, respectively (S2 Table). After combination of these two diabetes mellitus pathways, there were 75 unique CpGs corresponding to 27 genes which were used for further analyses. Among these 75 CpGs: 10 differentially methylated CpGs were in immediate proximity (200bp) of transcription start site (TSS); 5 were 1500 bp away from TSS (1500); 13 were in the 5’UTR; 6 were in the first exon; 29 CpGs were in gene bodies; and 12 were located in 3’UTR. The positions of the 75 CpGs probed in the Illumina 450K array is shown in S3 Table. Likewise, 28 genes (84 CpGs) and 19 genes (29 CpGs) associated with cognitive appraisal were selected from the type 1 and 2 diabetes mellitus pathway, respectively (S4 Table). There were 105 unique CpGs corresponding to 41 genes which were used for further analyses. Among the 105 CpGs: 11 differentially methylated CpGs were in immediate proximity (200bp) of transcription start site (TSS); 9 were 1500 bp away from TSS (1500); 18 were in the 5’UTR; 7 were in the first exon; 45 CpGs were in gene bodies; and 15 were located in 3’UTR. The positions of the 105 CpGs probed in Illumina 450K array are shown in S5 Table. Moreover, 100 of the 105 CpGs, methylation level and cognitive appraisal associations (87 significant and 13 non-significant) were similar when either the dichotomized or continuous (5-levels) versions of cognitive appraisal were entered into the Spearman analyses. The Spearman correlations are presented in S6 Table and illustrated in S1 Fig.

### Statistical analysis

First, we conducted Pearson Product-Moment correlations between C-peptide levels and predictors. Second, we performed mediation analyses and multiple corrections which have been described in our previous study [44].

**Fig 1:**
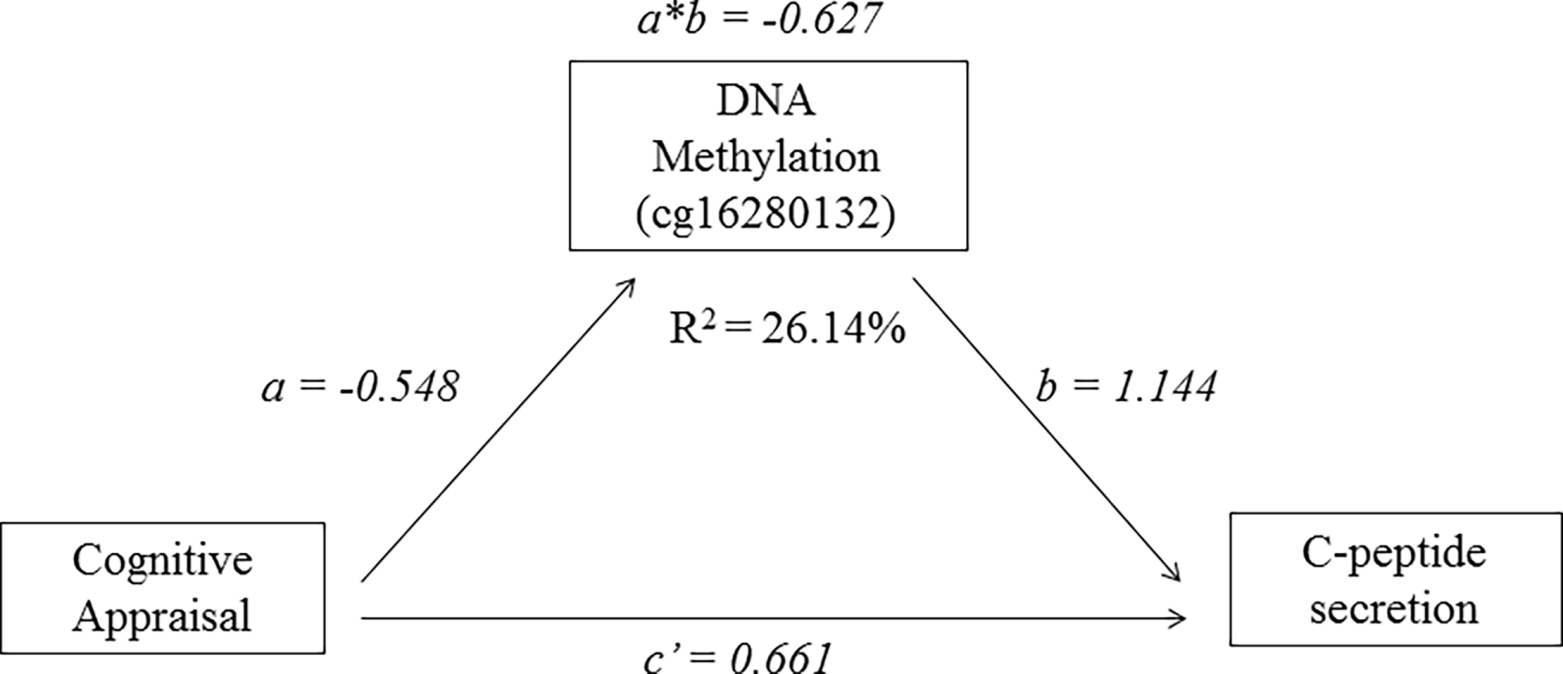
Mediation analysis on the relationship between exposure cognitive appraisal and C-peptide secretion. The effect of cognitive appraisal on the DNA methylation of cg16280132 from LTA is -0.548 (path a); the effect of the DNA methylation of cg16280132 on C-peptide level, controlling for cognitive appraisal is 1.144 (path b); the direct effect of cognitive appraisal on C-peptide level, controlling for the DNA methylation of cg16280132, is 0.661 (path c’); the mediating effect of cognitive appraisal on C-peptide level through DNA methylation of cg16280132 is -0.627 (path a*b). Together, the combination of cognitive appraisal and DNA methylation of cg16280132 explained 26.14% of the variance in C-peptide secretion.

To investigate whether the genes we selected mediated the relationship between exposure to PNMS and C-peptide level, we conducted mediation analyses using bootstrapping. The mediator model was a multiple regression model with C-peptide level as the outcome; objective hardship or cognitive appraisal as the PNMS variable of interest; and DNA methylation levels of CpGs as mediators. The theoretical model of the mediation analysis is presented in Fig 1. Path “*a*” is the effect of the predictor variable on the DNA methylation (mediator), path “*b*” is the effect of the DNA methylation on the outcome variable controlling for the predictor variable, and path “*c’*” is the direct effect of the predictor variable on the outcome variable controlling for DNA methylation (mediator). The coefficient “a*b” indicates the mediating effect of the predictor variable on the outcome variable through DNA methylation (mediator). We tested the indirect effects of PNMS on the two outcomes through each CpG site by computing 95% bias-corrected bootstrap confidence intervals, in accordance with Hayes [45]. The SPSS procedure PROCESS [45] was used to conduct the analyses. Each bootstrap resampled the initial sample 10000 times. In order to be able to recover the same results, the random seed for all bootstraps was fixed at 1,509,805,407 an integer randomly generated between 1 and 2,000,000,000 prior to the first bootstrap. A mediation effect was considered significant if 0 was not included in the bootstrap confidence interval.

To adjust significance criteria as a function of the number of analyses conducted for each metabolic outcome, the confidence interval ranges were corrected using Bonferroni. However, because two separate simple mediation analyses including two highly correlated mediators most likely lead to very similar results, traditional Bonferroni correction would be too conservative an approach. Thus, we used the method proposed by Li and Ji [46] to calculate the effective number (M_eff_) of independent tests, essentially reducing the denominator to a number that more accurately reflects the number of intercorrelated groups of CpG mediators. This method has been applied to our previous mediation analyses [44]. In order to apply this method to the current study, four steps were performed. For the *effect of cognitive appraisal on C-peptide level through 105 CpGs*: the first step was to calculate the correlations among the methylation levels of the 105 CpGs used in the mediation analyses; the CpG methylation levels were highly inter-correlated (|ρ| values: Min. = 0.00; Max. = 0.99; Median = 0.62). The second step was to compute the 105 eigenvalues from that correlation matrix. The third step was then to modify each eigenvalue following Li and Ji’s formula: *f*(*x*) = *I*(*x* ≥ 1) + (*x* − |*x*|). Basically, the new value’s integer portion is set to 0 if the eigenvalue was smaller than 1 and is set to 1 otherwise while the decimals remain unchanged. The last step consisted in summing the 105 new values to compute the effective number of independent tests (M_eff_) and use that M_eff_ as the correction factor in the Bonferroni method for multiple testing corrections. The resulting M_eff_ was 24. Therefore, the adjusted confidence intervals were recomputed for 24 tests at a 99.8% level using Bonferroni. For the effect of objective hardship through 75 CpGs: similar analyses were performed as described above. Among 75 CpGs, most of the CpG site methylation levels were highly correlated with each other (|ρ| values: Min. = 0.05; Max. = 0.99; Median = 0.85). The resulting M_eff_ was 12. Therefore, the adjusted confidence intervals were recomputed for 12 tests at a 99.6% level using Bonferroni.

To take into account the different variability in methylation across CpG sites, standardized mediation effects were also computed by standardizing the variables included in the model prior to running the analyses, that is, each variable was centered on the mean and then divided by its standard deviation. All the other effects presented in the mediation results section are unstandardized. All analyses were completed with SPSS 20.0 (SPSS, Chicago, IL).

## Results

### Participants’ characteristics

At the time of assessment, the children averaged 13.6 years of age (SD = 0.1) and 96.9% were from households in the middle class and above (lower class, 3.1%; middle class, 31.3%; upper-middle class, 53.1%; upper class, 12.5%). Table 1 shows minimum, maximum, mean and standard deviation for maternal variables, child variables and storm-related variables. No adolescents had diagnosed diabetes or G0≥126 mg/dL (the cutoff for diagnosis). Three adolescents had levels suggestive of prediabetes (100 to 125 mg/dL) (http://www.diabetes.org/diabetes-basics/diagnosis/).

**Table 1:** The characteristics of the mothers and children (N = 30).

	Mean	Std. Deviation	Minimum	Maximum
**Prenatal maternal stress (PNMS)**				
Objective hardship	11.10	4.26	5.00	21.00
Cognitive appraisal	0.70	0.47	0.00	1.00
**Mothers**				
SES	28.20	11.53	11.00	65.00
Life events (pregnancy)	5.70	2.93	1.00	12.00
Life events (13½ y)	3.41	2.34	0.00	9.00
GHQ anxiety (pregnancy)	0.25	0.26	0.00	0.86
GHQ anxiety (13½ y)	0.14	0.23	0.00	0.71
**Children's birth characteristics**				
Birth weight (g)	3400.33	667.39	1655.00	4432.00
Birth length (cm)	50.65	3.30	41.00	56.50
Birth ponderal index	26.29	3.58	20.20	34.52
Gestational age at birth (days)	98.83	96.64	-44.00	274.0
**Children at age 13**				
C-peptide secretion (μg/ml)	0.52	1.02	-2.47	4.33

Note: SES = socioeconomic status; GHQ = General Health Questionnaire

### Correlations between C-peptide secretion and predictors

As presented in Table 2, objective hardship was marginally significantly correlated with C-peptide secretion, with higher objective hardship predicting higher C-peptide secretion. Cognitive appraisal did not predict C-peptide secretion. Other predictors showed no significant associations with C-peptide secretion.

**Table 2:** Pearson correlations between C-peptide secretion and predictors.

Predictors	C-peptide secretion	
	R	p
Objective hardship (Storm32)	**.301**^**#**^	**.094**
Cognitive appraisal (Negative vs. Neutral/Positive)	.016	.935
SES	-.087	.635
Life events (pregnancy)	-.131	.474
Life events (13.5 y)	.202	.277
GHQ anxiety (pregnancy)	-.033	.859
GHQ anxiety (13.5 y)	-.062	.742
Birth weight	-.171	.357
Birth length	-.153	.410
Birth ponderal Index	-.128	.494
Gestational age	-.070	.703

Note.

^#^p<0.1

### Mediation analyses

#### Effect of cognitive appraisal

Our initial results indicated that 66 CpGs mapping to 25 genes negatively mediated the effect of cognitive appraisal on C-peptide secretion at a significance level of p < 0.05: negative cognitive appraisal predicted higher C-peptide secretion through DNA methylation (i.e., the value of coefficient a*b was negative) (Table 3). For 54 CpG sites corresponding to 17 genes, negative cognitive appraisal was associated with higher methylation levels (i.e., the values of coefficient *a* are negative), which were, in turn, associated with higher C-peptide production (i.e., the values of coefficient *b* are positive). For the remaining 12 CpG sites corresponding to 10 genes, negative cognitive appraisal was associated with lower methylation levels (i.e., the values of coefficient *a* are positive), which were in turn, associated with higher C-peptide production (i.e., the values of coefficient *b* are negative). This finding suggested that negative maternal cognitive appraisal could affect child C-peptide secretion via epigenetic changes, by recruiting different genes in the diabetes pathways. Interestingly, the highest negative mediating effects on C-peptide secretion were from CpGs located on *LTA*: from cg01157951 (a*b = -0.738) and, to a lesser extent, from cg16280132 (a*b = -0.627). Together, the combination of cognitive appraisal and DNA methylation explained between 5.66% and 31.95% of the variance in C-peptide secretion. After Bonferroni correction with confidence intervals at a 99.8% level, however, only one CpG site, cg16280132 from *LTA*, remained a significant mediator (Fig 1), with the model explaining 26.24% of the variance in C-peptide secretion (p< 0.05). The location of cg16280132 and that of the surrounding CpGs on *LTA* were shown in S2 Fig.

**Table 3:** Results of DNA methylation of genes that significantly mediated the effect of cognitive appraisal on C-peptide outcome in initial analyses. (The mediation effect that remained significant after multiple correction is highlighted in bold).

Gene	CpG site	CpG methylation				C-peptide Outcome							Mediation effect			
		R2^a^	Cognitive appraisal			R2^e^	CpG methylation			Cognitive appraisal						
			Effect (a)^b^	SE^c^	p-val.^d^		Effect (b)^f^	SE	p-val.	Effect (c')^g^	SE	p-val.	Effect (a*b)^h^	Boot SE^i^	Boot LLCI^j^	Boot ULCI^k^
ACSL3	cg22175006	0.1235#	0.8989	0.4526	0.0569	0.0566	-0.2168	0.1706	0.2147	0.2288	0.4364	0.6044	-0.1949	0.2257	-0.9453	-0.0058
ACSL6	cg14841483	0.2271**	-0.6234	0.2174	0.0078	0.2063*	0.8628	0.3259	0.0134	0.5717	0.4263	0.1910	-0.5378	0.3217	-1.4734	-0.1166
CD247	cg03727968	0.1572*	-0.4220	0.1846	0.0301	0.1195	0.7726	0.4040	0.0665	0.3600	0.4300	0.4099	-0.3260	0.2245	-1.0550	-0.0526
	cg07786657	0.2044*	-0.8620	0.3214	0.0121	0.0861	0.3766	0.2365	0.1229	0.3585	0.4509	0.4334	-0.3246	0.2288	-0.9924	-0.0501
	cg09032544	0.1347*	-0.4278	0.2049	0.0460	0.1258	0.7146	0.3628	0.0592	0.3396	0.4228	0.4289	-0.3057	0.2263	-1.0137	-0.0308
	cg09473725	0.0887	-0.3565	0.2159	0.1099	0.1836#	0.8194	0.3328	0.0205	0.3260	0.3982	0.4201	-0.2921	0.2373	-1.0609	-0.0110
	cg14278300	0.1966*	-0.4649	0.1776	0.0141	0.1822#	0.9923	0.4049	0.0210	0.4952	0.4245	0.2535	-0.4613	0.2930	-1.3508	-0.0904
CD3D	cg07728874	0.1769*	-0.5172	0.2108	0.0206	0.2607*	1.0002	0.3243	0.0047	0.5512	0.3987	0.1782	-0.5173	0.3139	-1.4693	-0.1124
	cg25643644	0.1078#	-0.4851	0.2638	0.0765	0.1615#	0.6289	0.2760	0.0308	0.3390	0.4078	0.4131	-0.3051	0.2344	-1.0684	-0.0270
	cg03074244	0.1851*	-0.6178	0.2449	0.0176	0.2144*	0.7805	0.2877	0.0115	0.5161	0.4131	0.2222	-0.4822	0.3039	-1.4196	-0.0986
	cg24841244	0.2174**	-0.8014	0.2874	0.0094	0.1607#	0.5759	0.2535	0.0313	0.4954	0.4357	0.2655	-0.4615	0.2998	-1.3382	-0.0912
CD3D; CD3G	cg03254928	0.1998*	-0.8943	0.3382	0.0133	0.0937	0.3734	0.2238	0.1068	0.3678	0.4477	0.4185	-0.3339	0.2365	-0.9794	-0.0594
	cg05160234	0.1966*	-0.6121	0.2339	0.0141	0.1865#	0.7624	0.3066	0.0194	0.5006	0.4233	0.2473	-0.4667	0.2876	-1.3408	-0.1006
	cg07545925	0.1369*	-0.5289	0.2509	0.0442	0.1986#	0.7333	0.2836	0.0154	0.4217	0.4054	0.3074	-0.3878	0.2715	-1.2389	-0.0544
	cg13750061	0.1672*	-0.5420	0.2286	0.0249	0.2546*	0.9113	0.3002	0.0053	0.5278	0.3980	0.1959	-0.4939	0.3161	-1.4554	-0.0905
CD3E	cg06164961	0.2148**	-0.8648	0.3125	0.0099	0.1988#	0.5891	0.2278	0.0154	0.5434	0.4250	0.2119	-0.5094	0.3000	-1.4046	-0.1229
FASLG	cg06983746	0.2076*	-0.7375	0.2723	0.0114	0.1386	0.5642	0.2710	0.0469	0.4500	0.4386	0.3140	-0.4161	0.2774	-1.2529	-0.0870
	cg10161121	0.2151**	-0.8385	0.3028	0.0098	0.1309	0.4932	0.2448	0.0540	0.4475	0.4427	0.3210	-0.4136	0.2761	-1.1779	-0.0785
	cg00071250	0.1416*	-0.3416	0.1590	0.0404	0.1764#	1.0907	0.4539	0.0234	0.4066	0.4121	0.3326	-0.3726	0.2902	-1.3099	-0.0306
HLA-DMB	cg01374870	0.1931*	0.7172	0.2771	0.0151	0.1229	-0.5222	0.2687	0.0625	0.4085	0.4386	0.3600	-0.3745	0.2598	-1.1986	-0.0699
	cg10411221	0.1606*	0.3615	0.1561	0.0282	0.1227	-0.9258	0.4769	0.0627	0.3685	0.4301	0.3990	-0.3346	0.2555	-1.1509	-0.0386
	cg13524037	0.1442*	0.4692	0.2161	0.0385	0.1333	-0.6974	0.3426	0.0517	0.3612	0.4233	0.4011	-0.3273	0.2453	-1.1294	-0.0437
HLA-DQA1	cg03787837	0.1482*	0.6957	0.3152	0.0357	0.0765	-0.3620	0.2424	0.1470	0.2857	0.4380	0.5197	-0.2518	0.2032	-0.9221	-0.0328
HLA-DRB1	cg17376015	0.1472*	1.0956	0.4983	0.0363	0.1098	-0.2744	0.1505	0.0794	0.3346	0.4298	0.4431	-0.3007	0.2296	-1.1093	-0.0335
HLA-E	cg09569347	0.1118#	-0.3096	0.1649	0.0710	0.2121*	1.1529	0.4278	0.0120	0.3909	0.3962	0.3326	-0.3569	0.2546	-1.1486	-0.0386
	cg16535080	0.1681*	-0.5213	0.2192	0.0244	0.0768	0.5215	0.3485	0.1462	0.3058	0.4432	0.4961	-0.2718	0.1736	-0.8152	-0.0514
	cg20652371	0.1657*	-0.3534	0.1499	0.0256	0.0805	0.7809	0.5087	0.1365	0.3099	0.4417	0.4889	-0.2760	0.2078	-0.9392	-0.0306
	cg21366673	0.1771*	-0.5371	0.2188	0.0206	0.1334	0.6888	0.3382	0.0516	0.4039	0.4317	0.3578	-0.3700	0.2586	-1.1616	-0.0507
	cg27486585	0.1784*	-0.8781	0.3561	0.0201	0.0867	0.3410	0.2134	0.1216	0.3334	0.4436	0.4588	-0.2995	0.2131	-0.9007	-0.0557
IL1RAP	cg16588163	0.1584*	0.3515	0.1531	0.0294	0.1397	-1.0074	0.4816	0.0460	0.3880	0.4253	0.3697	-0.3541	0.2677	-1.2150	-0.0527
IRF1	cg15375424	0.1903*	-0.7429	0.2896	0.0159	0.0956	0.4406	0.2611	0.1030	0.3612	0.4446	0.4236	-0.3273	0.2224	-0.9539	-0.0657
LTA	cg00501919	0.1389*	-0.3534	0.1663	0.0425	0.1936#	1.0923	0.4293	0.0170	0.4199	0.4071	0.3114	-0.3860	0.3312	-1.4865	-0.0282
	cg01157951	0.2629**	-0.5945	0.1882	0.0038	0.3195**	1.2405	0.3486	0.0014	0.7715	0.4042	0.0670	-0.7376	0.3856	-1.7425	-0.1840
	cg13815684	0.2299**	-0.7098	0.2455	0.0073	0.2304*	0.8072	0.2841	0.0084	0.6068	0.4205	0.1606	-0.5729	0.3612	-1.5791	-0.0916
	cg14441276	0.3003**	-0.8618	0.2486	0.0017	0.1200	0.5750	0.3000	0.0659	0.5294	0.4718	0.2716	-0.4955	0.3788	-1.6595	-0.0595
	**cg16280132**	0.2396**	**-0.5480**	**0.1845**	**0.0060**	0.2614*	**1.1444**	**0.3703**	**0.0046**	**0.6610**	**0.4146**	**0.1225**	**-0.6271**	**0.3613**	**-1.6152**	**-0.1293**
	cg17709873	0.1972*	-0.9083	0.3464	0.0140	0.0620	0.2964	0.2223	0.1936	0.3031	0.4547	0.5107	-0.2692	0.2631	-0.9017	-0.0569
	cg22318806	0.2564**	-0.6815	0.2193	0.0043	0.1447	0.7159	0.3352	0.0419	0.5218	0.4512	0.2576	-0.4879	0.3297	-1.3733	-0.0986
	cg26348243	0.2320**	-0.7874	0.2707	0.0070	0.2191*	0.7140	0.2595	0.0105	0.5961	0.4242	0.1714	-0.5622	0.3500	-1.5722	-0.1186
	cg02402436	0.2721**	-0.9882	0.3054	0.0031	0.1768#	0.5683	0.2362	0.0232	0.5955	0.4474	0.1943	-0.5616	0.3551	-1.6889	-0.1304
	cg09621572	0.2576**	-0.9424	0.3023	0.0042	0.1954#	0.6036	0.2359	0.0164	0.6027	0.4380	0.1801	-0.5688	0.3511	-1.5937	-0.1213
	cg10476003	0.3185**	-1.0105	0.2793	0.0012	0.1243	0.5208	0.2664	0.0610	0.5602	0.4769	0.2504	-0.5263	0.3269	-1.4630	-0.1279
	cg11586857	0.3423***	-0.7631	0.1999	0.0007	0.1551	0.8133	0.3656	0.0346	0.6545	0.4768	0.1811	-0.6206	0.3643	-1.7099	-0.1655
	cg14437551	0.2572**	-1.1029	0.3542	0.0042	0.1837#	0.4994	0.2028	0.0204	0.5847	0.4410	0.1960	-0.5508	0.3607	-1.6776	-0.1372
	cg14597739	0.3055**	-1.2493	0.3560	0.0015	0.1320	0.4212	0.2081	0.0529	0.5601	0.4703	0.2440	-0.5262	0.3379	-1.4944	-0.0851
	cg16219283	0.3076**	-1.0178	0.2886	0.0015	0.1404	0.5359	0.2554	0.0454	0.5794	0.4687	0.2271	-0.5455	0.3597	-1.6102	-0.0990
	cg17169196	0.2449**	-0.7914	0.2626	0.0054	0.1938#	0.6920	0.2718	0.0169	0.5816	0.4347	0.1921	-0.5477	0.3512	-1.5936	-0.1101
	cg21999229	0.3116**	-1.1934	0.3353	0.0013	0.1777#	0.5191	0.2150	0.0228	0.6535	0.4598	0.1667	-0.6195	0.3759	-1.7185	-0.1427
MAP3K14	cg16826777	0.1422*	0.3932	0.1825	0.0399	0.1797#	-0.9590	0.3946	0.0220	0.4110	0.4114	0.3266	-0.3771	0.2720	-1.2359	-0.0627
MAPK14	cg20559215	0.2232**	-0.7066	0.2491	0.0084	0.2576*	0.8413	0.2749	0.0050	0.6283	0.4112	0.1382	-0.5944	0.3533	-1.5512	-0.0921
NFKBIA	cg00689225	0.1074#	-0.4146	0.2259	0.0771	0.1580#	0.7264	0.3229	0.0328	0.3351	0.4086	0.4193	-0.3012	0.2753	-1.2156	-0.0051
PIK3CD	cg01320698	0.1012#	-0.2340	0.1317	0.0866	0.1075	1.0268	0.5701	0.0829	0.2742	0.4192	0.5187	-0.2402	0.2220	-0.9956	-0.0005
	cg07499142	0.1577*	-0.4975	0.2173	0.0298	0.1389	0.7078	0.3395	0.0467	0.3860	0.4254	0.3722	-0.3521	0.2545	-1.1561	-0.0610
	cg07970040	0.1951*	-0.6844	0.2627	0.0145	0.2125*	0.7243	0.2685	0.0119	0.5296	0.4161	0.2139	-0.4957	0.2927	-1.3738	-0.1201
PIK3R6	cg00409104	0.0890	0.2631	0.1590	0.1092	0.1648#	-1.0537	0.4569	0.0290	0.3111	0.4028	0.4466	-0.2772	0.2273	-1.0400	-0.0158
PRF1	cg12433559	0.1883*	-0.3493	0.1371	0.0166	0.1340	1.1024	0.5398	0.0510	0.4190	0.4345	0.3435	-0.3851	0.2545	-1.1864	-0.0684
PRKAG2	cg22528270	0.2450**	1.1694	0.3879	0.0054	0.1587#	-0.4240	0.1880	0.0324	0.5297	0.4441	0.2433	-0.4958	0.3323	-1.4525	-0.0590
PRKCH	cg14001486	0.2173**	-0.5379	0.1929	0.0094	0.1732#	0.8906	0.3747	0.0248	0.5130	0.4324	0.2458	-0.4790	0.3125	-1.4473	-0.0987
	cg17306848	0.1452*	-0.6159	0.2824	0.0377	0.1900#	0.6373	0.2534	0.0182	0.4264	0.4095	0.3070	-0.3925	0.2979	-1.3321	-0.0310
PRKCZ	cg00300046	0.2051*	-0.5455	0.2030	0.0120	0.0776	0.5661	0.3761	0.1439	0.3428	0.4531	0.4560	-0.3088	0.2646	-1.1748	-0.0481
	cg02481000	0.3046**	0.6206	0.1772	0.0016	0.1060	-0.7582	0.4242	0.0852	0.5044	0.4770	0.2996	-0.4705	0.3484	-1.5189	-0.0580
	cg10334053	0.1782*	-0.6657	0.2702	0.0202	0.0825	0.4386	0.2818	0.1313	0.3259	0.4445	0.4698	-0.2919	0.2022	-0.9141	-0.0499
SOCS1	cg03014241	0.1314*	-0.3527	0.1713	0.0490	0.1082	0.7922	0.4382	0.0818	0.3133	0.4263	0.4687	-0.2794	0.2368	-1.0342	-0.0062
TNFRSF1B	cg05599723	0.2262**	0.5913	0.2066	0.0079	0.1026	-0.6396	0.3645	0.0906	0.4121	0.4530	0.3711	-0.3782	0.2577	-1.1671	-0.0791
	cg15526535	0.0855	-0.2585	0.1598	0.1169	0.0717	0.6912	0.4793	0.1608	0.2126	0.4238	0.6200	-0.1787	0.1373	-0.6035	-0.0044
	cg22677556	0.1583*	-0.5670	0.2471	0.0294	0.1934#	0.7348	0.2890	0.0170	0.4506	0.4118	0.2836	-0.4166	0.2613	-1.2355	-0.0885

Note:

^#^p<0.1

*p<0.05

**p<0.01

***p<0.001

^a^: R^2^ for path a

^b^: Unstandardized coefficient of maternal cognitive appraisal on CpG Methylation (effect *a*)

^c^: Standard error of the coefficient

^d^: p-value of the coefficient

^e^: R^2^ for paths b + c'

^f^: Unstandardized coefficient of methylation on C-peptide outcome, controlling for maternal cognitive appraisal (effect *b*)

^g^: Unstandardized effect of maternal cognitive appraisal on C-peptide outcome, controlling for DNA methylation (path *c'*)

^h^: Mediation effect based on unstandardized coefficients

^i^: Bootstrapping Standard error of the indirect effect

^j^: Mediation effect's bootstrapping lower level confidence interval

^k^: Mediation effect's bootstrapping upper level confidence interval

#### Effect of objective hardship

Our analyses indicated that 61 CpGs, mapping to 23 genes, positively mediated the effect of objective hardship on C-peptide secretion: higher objective hardship predicted higher C-peptide through DNA methylation (i.e., the value of coefficient a*b is positive) (S7 Table). For 51 CpG sites corresponding to 15 genes, higher objective hardship exposure was related to higher DNA methylation (i.e., the value of coefficient *a* is positive), which in turn, was associated with higher C-peptide production (i.e., the value of coefficient *b* is positive). For the remaining 10 CpG sites, corresponding to 8 genes, higher objective hardship exposure was related to lower DNA methylation (i.e., the value of coefficient *a* is negative), which were in turn, associated with higher C-peptide secretion (i.e., the value of coefficient *b* is negative). The highest positive mediating effect of DNA methylation (a*b = 0.072) on C-peptide production involved the cg16280132 CpG site located on *LTA* in a model explaining 22.9% of the variance in C-peptide (p < 0.05). Together, the combination of objective hardship and DNA methylation explained between 8.83% and 24.10% of the variance in C-peptide secretion. However, none of the CpGs associated with C-peptide secretion remained significant mediators after Bonferroni correction with confidence intervals at a 99.6% level.

## Discussion

The main objective of the current study was to determine whether DNA methylation mediated the association between disaster-related maternal objective hardship and cognitive appraisal and C-peptide secretion in the offspring. In this study we found that adolescents whose mothers experienced higher objective hardship exhibited higher C-peptide secretion in response to an oral glucose tolerance test (Table 2). Furthermore, DNA methylation of type 1 and 2 diabetes-related genes had a positive mediation effect, triggered by objective hardship, on C-peptide secretion (S7 Table). The combination of objective hardship and DNA methylation explained up to 24.10% of the variance in C-peptide secretion. As for maternal cognitive appraisal of the ice storm, DNA methylation of type 1 and 2 diabetes-related genes had a negative mediation effect, triggered by cognitive appraisal, on C-peptide secretion (Table 3). The combination of cognitive appraisal and DNA methylation explained up to 31.95% of the variance in C-peptide secretion. As such, both a negative cognitive appraisal and higher objective hardship predicted higher C-peptide secretion through DNA methylation. However, only one CpG site (cg16280132) from *LTA* remained a significant mediator between cognitive appraisal and C-peptide secretion after conservative Bonferroni multiple corrections were conducted.

We observed that the severity of objective hardship was associated with C-peptide secretion (at the trend level) in the children. Together with our previous study on the effect of objective hardship on insulin secretion [29], we conclude that children whose mothers experienced higher objective hardship exhibited both higher insulin and C-peptide secretion in response to the oral glucose tolerance test. This finding is in accordance with those from both animal [47] and human [18] studies demonstrating that higher stress levels during pregnancy were associated with adverse metabolic outcomes in offspring. Our mediation findings suggest a great number of possible mediation effects from objective hardship and cognitive appraisal that were significant at the test-wise level, but they did not survive correction for multiple testing at the experiment-wise level given the limited sample size. Although there was not a significant correlation between cognitive appraisal and C-peptide secretion, we did find a significant indirect effect (mediation effect) of cognitive appraisal on C-peptide secretion via DNA methylation, even after the multiple corrections.

The effects of PNMS reflect a number of interacting mechanistic pathways [48–50], including epigenetics [50]. Given the important role of type 1 and 2 diabetes mellitus pathways on outcomes such as obesity, insulin resistance and diabetes, we selected both type 1 and 2 diabetes-related sets of genes in which methylation levels were associated with objective hardship or cognitive appraisal to conduct the mediation analyses. Despite no observed direct relationship between cognitive appraisal and C-peptide levels, we performed mediation analyses with DNA methylation of selected CpGs which were observed to be related to cognitive appraisal since mediation analyses do not require a significant association between predictor and outcome [45]. Among the selected genes, *LTA* possessed the only CpG site (cg16280132) which survived following multiple correction and showed one of the highest mediation effects in our analyses. Moreover, *LTA* had the greatest number of CpGs in which the DNA methylation levels mediated the effects of cognitive appraisal and objective hardship on C-peptide secretion. Interestingly, in our previous studies *LTA* was also found to mediate the effect of objective hardship on BMI, central adiposity and cytokine secretion [33, 34], and the effect of cognitive appraisal on BMI and central adiposity [35]. LTA is a member of the tumor necrosis factor family that is involved in the formation of secondary lymphoid organs during development, and plays a role in apoptosis [51]. Although no direct association between methylation of *LTA* and metabolic outcomes has been reported, various studies have investigated the relationship between the *LTA* polymorphism and insulin resistance and type 1 and 2 mellitus diabetes. For instance, the *NcoI* polymorphism, a polymorphic site that is located in the first intron of the *LTA*, was found to decrease insulin resistance in Japanese men [52]. In addition, the *NcoI* polymorphism was reported to be involved in hypertriglyceridemia, one component of insulin resistance syndrome, in Spanish patients with type 2 diabetes [53]. Likewise, the association between *NcoI* polymorphism of *LTA* and the risk factor hyperinsulinaemia was reported in patients with coronary artery disease [54]. Finally, in a study to investigate the impact of *LTA* polymorphism on serum parameters related to metabolic disorders in patients with coronary artery disease (CAD), an association of *LTA* alleles with the risk factor hyperinsulinaemia was reported, suggesting that *LTA* may contribute to the complex susceptibility for metabolic syndrome in patients with CAD [55]. Together, given the above evidence on the role of *LTA* on metabolic outcomes, methylation changes on *LTA* could be considered an important mechanism through which PNMS could affect C-peptide secretion.

The present results are somewhat limited by the small sample size. Moreover, we did not obtain RNA samples due to the low amount of blood collected, and as such, the consequences of these methylation changes and the alteration of downstream gene expression could not be verified, an issue that should be addressed in further studies. Given the tissue and cell-specificity of epigenetics, we isolated T cells from whole blood in order to minimize the heterogeneity of the cell population; however, the heterogeneity between blood and pancreas tissue is a limitation of our study. In addition, we cannot exclude the potential effects of postnatal environmental factors on the modification of DNA methylation profiles in childhood. Moreover, perhaps due to the small sample size, the mediation effect of many CpGs did not resist the conservative Bonferroni correction for multiple comparisons. Therefore, further research with a larger sample is warranted to more precisely define the points as noted above. However, the use of bootstrapping procedures in the mediation analyses is a powerful approach because it takes into account the fact that the sampling distribution of the mediated effect is skewed away from 0 allowing it to be applied to small-to-moderate samples.

This is the first study to demonstrate that DNA methylation mediates the relationship between maternal cognitive appraisal of a natural disaster in pregnancy and C-peptide production in her offspring during adolescence. Our study was able to identify epigenetic mechanisms associated specifically with maternal objective hardship and cognitive appraisal, and metabolic outcomes in the offspring. This study suggests that maternal stress could represent one factor contributing to dysfunctions related to metabolic outcomes such as insulin resistance and type 1 or type 2 diabetes mellitus among offspring.

## Conclusions

These data highlight the important role of DNA methylation of diabetes-related genes on mediating the relationship between specific aspects of prenatal maternal stress and metabolic outcomes in offspring. Our findings add new evidence on the critical role of fetal programming through epigenetic mechanisms in response to environmental factors.

## Supporting information

S1 Fig Scatterplots demonstrating the relationship between CpG methylation and cognitive appraisal (5-levels). (DOCX)

S2 Fig The map of LTA gene with 19 CpG locations. (DOCX)

S1 Table Raw data concerning objective hardship, cognitive appraisal, life events, GHQ scores, SES and C-peptide secretion. (XLSX)

S2 Table CpGs associated with objective hardship were selected from the type 1 and 2 diabetes mellitus pathway (The overlapping CpGs were highlighted in bold). (XLSX)

S3 Table Locations of 75 CpGs associated with objective hardship. (XLSX)

S4 Table CpGs associated with cognitive appraisal were selected from the type 1 and 2 diabetes mellitus pathway (The overlapping CpGs were highlighted in bold). (XLSX)

S5 Table Locations of 105 CpGs associated with cognitive appraisal. (XLSX)

S6 Table Spearman rank correlation coefficients for the associations between CpG methylations levels and cognitive appraisal (2-level and 5-level) for the full sample (34 participants from Cao-Lei et al. 2015) and the sample (30 participants) used in the present study. (DOCX)

S7 Table DNA methylation of genes mediated the effect of objective hardship on C-peptide Outcome. (XLSX)
