# Supplementary figures and images for: DNA methylation mediates the effect of maternal cognitive appraisal of a disaster in pregnancy on the child’s C-peptide secretion in adolescence: Project Ice Storm
Source: PLoS One. 2018 Feb 5;13(2):e0192199. doi: 10.1371/journal.pone.0192199 (PMC5798828; doi:10.1371/journal.pone.0192199)

**S2 Fig. The map of LTA gene with 19 CpG locations.**


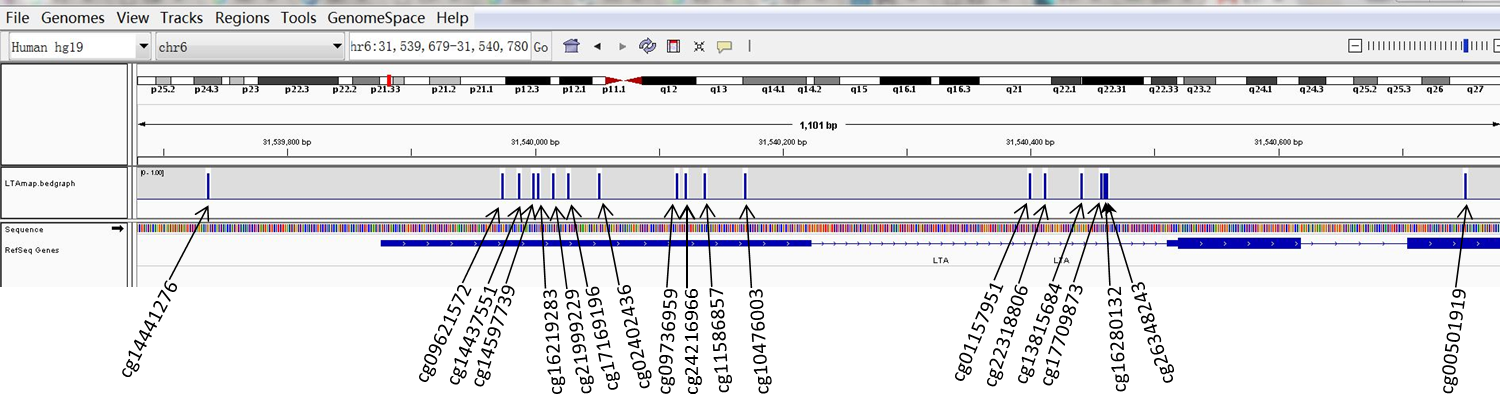

Supplement: S2 Fig — (DOCX) [file pone.0192199.s002.docx]
