# Supplementary material for: DNA methylation mediates the effect of maternal cognitive appraisal of a disaster in pregnancy on the child’s C-peptide secretion in adolescence: Project Ice Storm
Source: PLoS One. 2018 Feb 5;13(2):e0192199. doi: 10.1371/journal.pone.0192199 (PMC5798828; doi:10.1371/journal.pone.0192199)
Supplement: S6 Table — (DOCX) [file pone.0192199.s008.docx]

**S6 Table. Spearman rank correlation coefficients for the associations between CpG methylations levels and cognitive appraisal (2-level and 5-level) for the full sample (34 participants from Cao-Lei et al. 2015) and the sample (30 participants) used in the present study.**

|  | Cognitive Appraisal (34 participants) | | | |  |  | Cognitive Appraisal (30 participants) | | | |
| --- | --- | --- | --- | --- | --- | --- | --- | --- | --- | --- |
| CpGs | 5-levels | | 2-levels | |  |  | 5-levels | | 2-levels | |
|  | Spearman's rho | p | Spearman's rho | p |  |  | Spearman's rho | p | Spearman's rho | p |
| cg13642872 | -0.316 | 0.07 | -0.464 | 0.01 |  |  | -0.301 | 0.11 | -0.483 | 0.01 |
| cg22175006 | 0.436 | 0.01 | 0.514 | 0.00 |  |  | 0.350 | 0.06 | 0.441 | 0.01 |
| cg14841483 | -0.443 | 0.01 | -0.477 | 0.00 |  |  | -0.410 | 0.02 | -0.450 | 0.01 |
| cg03727968 | -0.443 | 0.01 | -0.458 | 0.01 |  |  | -0.398 | 0.03 | -0.416 | 0.02 |
| cg07786657 | -0.443 | 0.01 | -0.483 | 0.00 |  |  | -0.390 | 0.03 | -0.433 | 0.02 |
| cg09032544 | -0.434 | 0.01 | -0.439 | 0.01 |  |  | -0.368 | 0.05 | -0.366 | 0.05 |
| cg09473725 | -0.328 | 0.06 | -0.351 | 0.04 |  |  | -0.265 | 0.16 | -0.282 | 0.13 |
| cg13210595 | -0.367 | 0.03 | -0.326 | 0.06 |  |  | -0.331 | 0.07 | -0.273 | 0.14 |
| cg14278300 | -0.449 | 0.01 | -0.477 | 0.00 |  |  | -0.399 | 0.03 | -0.424 | 0.02 |
| cg24336674 | -0.320 | 0.07 | -0.339 | 0.05 |  |  | -0.241 | 0.20 | -0.240 | 0.20 |
| cg07728874 | -0.441 | 0.01 | -0.445 | 0.01 |  |  | -0.406 | 0.03 | -0.399 | 0.03 |
| cg25643644 | -0.340 | 0.05 | -0.376 | 0.03 |  |  | -0.292 | 0.12 | -0.324 | 0.08 |
| cg03074244 | -0.411 | 0.02 | -0.427 | 0.01 |  |  | -0.394 | 0.03 | -0.408 | 0.03 |
| cg24841244 | -0.438 | 0.01 | -0.464 | 0.01 |  |  | -0.393 | 0.03 | -0.416 | 0.02 |
| cg03254928 | -0.435 | 0.01 | -0.458 | 0.01 |  |  | -0.372 | 0.04 | -0.399 | 0.03 |
| cg05160234 | -0.443 | 0.01 | -0.483 | 0.00 |  |  | -0.374 | 0.04 | -0.416 | 0.02 |
| cg07545925 | -0.390 | 0.02 | -0.401 | 0.02 |  |  | -0.339 | 0.07 | -0.340 | 0.07 |
| cg13750061 | -0.418 | 0.01 | -0.439 | 0.01 |  |  | -0.378 | 0.04 | -0.399 | 0.03 |
| cg06164961 | -0.464 | 0.01 | -0.483 | 0.00 |  |  | -0.438 | 0.02 | -0.458 | 0.01 |
| cg24612198 | -0.292 | 0.09 | -0.301 | 0.08 |  |  | -0.209 | 0.27 | -0.214 | 0.26 |
| cg15880738 | -0.352 | 0.04 | -0.370 | 0.03 |  |  | -0.253 | 0.18 | -0.256 | 0.17 |
| cg06983746 | -0.479 | 0.00 | -0.527 | 0.00 |  |  | -0.409 | 0.02 | -0.458 | 0.01 |
| cg10161121 | -0.467 | 0.01 | -0.496 | 0.00 |  |  | -0.422 | 0.02 | -0.450 | 0.01 |
| cg00071250 | -0.464 | 0.01 | -0.477 | 0.00 |  |  | -0.391 | 0.03 | -0.391 | 0.03 |
| cg03812172 | 0.325 | 0.06 | 0.383 | 0.03 |  |  | 0.258 | 0.17 | 0.324 | 0.08 |
| cg06422189 | -0.568 | 0.00 | -0.540 | 0.00 |  |  | -0.549 | 0.00 | -0.508 | 0.00 |
| cg08245665 | 0.322 | 0.06 | 0.420 | 0.01 |  |  | 0.280 | 0.13 | 0.416 | 0.02 |
| cg11187245 | -0.465 | 0.01 | -0.521 | 0.00 |  |  | -0.354 | 0.06 | -0.408 | 0.03 |
| cg22731440 | -0.449 | 0.01 | -0.427 | 0.01 |  |  | -0.374 | 0.04 | -0.332 | 0.07 |
| cg01374870 | 0.415 | 0.01 | 0.483 | 0.00 |  |  | 0.337 | 0.07 | 0.408 | 0.03 |
| cg10411221 | 0.388 | 0.02 | 0.508 | 0.00 |  |  | 0.288 | 0.12 | 0.433 | 0.02 |
| cg10453850 | 0.363 | 0.04 | 0.389 | 0.02 |  |  | 0.283 | 0.13 | 0.298 | 0.11 |
| cg13524037 | 0.363 | 0.04 | 0.464 | 0.01 |  |  | 0.265 | 0.16 | 0.382 | 0.04 |
| cg26964592 | 0.244 | 0.16 | 0.320 | 0.07 |  |  | 0.167 | 0.38 | 0.256 | 0.17 |
| cg02920196 | 0.373 | 0.03 | 0.339 | 0.05 |  |  | 0.289 | 0.12 | 0.223 | 0.24 |
| cg03787837 | 0.451 | 0.01 | 0.439 | 0.01 |  |  | 0.434 | 0.02 | 0.424 | 0.02 |
| cg22933800 | -0.322 | 0.06 | -0.257 | 0.14 |  |  | -0.272 | 0.15 | -0.172 | 0.36 |
| cg00995368 | -0.376 | 0.03 | -0.433 | 0.01 |  |  | -0.231 | 0.22 | -0.298 | 0.11 |
| cg03344051 | 0.257 | 0.14 | 0.213 | 0.23 |  |  | 0.297 | 0.11 | 0.240 | 0.20 |
| cg21493951 | -0.385 | 0.02 | -0.339 | 0.05 |  |  | -0.339 | 0.07 | -0.248 | 0.19 |
| cg23214071 | -0.386 | 0.02 | -0.307 | 0.08 |  |  | -0.294 | 0.11 | -0.181 | 0.34 |
| cg23464743 | 0.365 | 0.03 | 0.339 | 0.05 |  |  | 0.339 | 0.07 | 0.282 | 0.13 |
| cg04601775 | 0.373 | 0.03 | 0.314 | 0.07 |  |  | 0.323 | 0.08 | 0.248 | 0.19 |
| cg17316649 | 0.456 | 0.01 | 0.439 | 0.01 |  |  | 0.525 | 0.00 | 0.517 | 0.00 |
| cg17376015 | 0.454 | 0.01 | 0.401 | 0.02 |  |  | 0.381 | 0.04 | 0.290 | 0.12 |
| cg05201185 | -0.257 | 0.14 | -0.314 | 0.07 |  |  | -0.153 | 0.42 | -0.214 | 0.26 |
| cg09569347 | -0.357 | 0.04 | -0.370 | 0.03 |  |  | -0.303 | 0.10 | -0.307 | 0.10 |
| cg13036546 | -0.367 | 0.03 | -0.414 | 0.01 |  |  | -0.255 | 0.17 | -0.298 | 0.11 |
| cg16535080 | -0.363 | 0.03 | -0.439 | 0.01 |  |  | -0.275 | 0.14 | -0.357 | 0.05 |
| cg17615629 | -0.362 | 0.04 | -0.383 | 0.03 |  |  | -0.269 | 0.15 | -0.282 | 0.13 |
| cg20652371 | -0.428 | 0.01 | -0.452 | 0.01 |  |  | -0.355 | 0.05 | -0.374 | 0.04 |
| cg21366673 | -0.424 | 0.01 | -0.477 | 0.00 |  |  | -0.364 | 0.05 | -0.424 | 0.02 |
| cg27486585 | -0.435 | 0.01 | -0.458 | 0.01 |  |  | -0.381 | 0.04 | -0.399 | 0.03 |
| cg03298800 | -0.352 | 0.04 | -0.376 | 0.03 |  |  | -0.246 | 0.19 | -0.273 | 0.14 |
| cg08173915 | 0.481 | 0.00 | 0.546 | 0.00 |  |  | 0.423 | 0.02 | 0.492 | 0.01 |
| cg22669060 | 0.311 | 0.07 | 0.326 | 0.06 |  |  | 0.186 | 0.32 | 0.189 | 0.32 |
| cg06392753 | 0.327 | 0.06 | 0.383 | 0.03 |  |  | 0.180 | 0.34 | 0.240 | 0.20 |
| cg16588163 | 0.407 | 0.02 | 0.445 | 0.01 |  |  | 0.359 | 0.05 | 0.399 | 0.03 |
| cg15375424 | -0.444 | 0.01 | -0.477 | 0.00 |  |  | -0.405 | 0.03 | -0.433 | 0.02 |
| cg02405213 | -0.205 | 0.24 | -0.289 | 0.10 |  |  | -0.137 | 0.47 | -0.248 | 0.19 |
| cg00501919 | -0.469 | 0.01 | -0.427 | 0.01 |  |  | -0.375 | 0.04 | -0.315 | 0.09 |
| cg01157951 | -0.518 | 0.00 | -0.558 | 0.00 |  |  | -0.447 | 0.01 | -0.492 | 0.01 |
| cg13815684 | -0.553 | 0.00 | -0.583 | 0.00 |  |  | -0.475 | 0.01 | -0.500 | 0.00 |
| cg14441276 | -0.535 | 0.00 | -0.590 | 0.00 |  |  | -0.458 | 0.01 | -0.517 | 0.00 |
| cg16280132 | -0.498 | 0.00 | -0.502 | 0.00 |  |  | -0.475 | 0.01 | -0.483 | 0.01 |
| cg17709873 | -0.517 | 0.00 | -0.546 | 0.00 |  |  | -0.461 | 0.01 | -0.500 | 0.00 |
| cg22318806 | -0.566 | 0.00 | -0.583 | 0.00 |  |  | -0.497 | 0.01 | -0.508 | 0.00 |
| cg26348243 | -0.504 | 0.00 | -0.508 | 0.00 |  |  | -0.471 | 0.01 | -0.475 | 0.01 |
| cg02402436 | -0.496 | 0.00 | -0.546 | 0.00 |  |  | -0.414 | 0.02 | -0.466 | 0.01 |
| cg09621572 | -0.552 | 0.00 | -0.577 | 0.00 |  |  | -0.494 | 0.01 | -0.508 | 0.00 |
| cg10476003 | -0.591 | 0.00 | -0.627 | 0.00 |  |  | -0.508 | 0.00 | -0.542 | 0.00 |
| cg11586857 | -0.603 | 0.00 | -0.640 | 0.00 |  |  | -0.531 | 0.00 | -0.567 | 0.00 |
| cg14437551 | -0.505 | 0.00 | -0.527 | 0.00 |  |  | -0.428 | 0.02 | -0.441 | 0.01 |
| cg14597739 | -0.566 | 0.00 | -0.615 | 0.00 |  |  | -0.474 | 0.01 | -0.525 | 0.00 |
| cg16219283 | -0.537 | 0.00 | -0.602 | 0.00 |  |  | -0.475 | 0.01 | -0.550 | 0.00 |
| cg17169196 | -0.504 | 0.00 | -0.552 | 0.00 |  |  | -0.430 | 0.02 | -0.475 | 0.01 |
| cg21999229 | -0.551 | 0.00 | -0.602 | 0.00 |  |  | -0.486 | 0.01 | -0.542 | 0.00 |
| cg16826777 | 0.429 | 0.01 | 0.483 | 0.00 |  |  | 0.335 | 0.07 | 0.391 | 0.03 |
| cg05093818 | -0.250 | 0.15 | -0.176 | 0.32 |  |  | -0.265 | 0.16 | -0.181 | 0.34 |
| cg20559215 | -0.547 | 0.00 | -0.583 | 0.00 |  |  | -0.473 | 0.01 | -0.517 | 0.00 |
| cg00689225 | -0.343 | 0.05 | -0.364 | 0.03 |  |  | -0.288 | 0.12 | -0.307 | 0.10 |
| cg01320698 | -0.377 | 0.03 | -0.383 | 0.03 |  |  | -0.286 | 0.13 | -0.282 | 0.13 |
| cg07499142 | -0.400 | 0.02 | -0.427 | 0.01 |  |  | -0.335 | 0.07 | -0.357 | 0.05 |
| cg07970040 | -0.419 | 0.01 | -0.427 | 0.01 |  |  | -0.357 | 0.05 | -0.357 | 0.05 |
| cg04610450 | 0.285 | 0.10 | 0.326 | 0.06 |  |  | 0.183 | 0.33 | 0.223 | 0.24 |
| cg11953794 | 0.338 | 0.05 | 0.339 | 0.05 |  |  | 0.288 | 0.12 | 0.273 | 0.14 |
| cg25342409 | -0.328 | 0.06 | -0.295 | 0.09 |  |  | -0.203 | 0.28 | -0.147 | 0.44 |
| cg00409104 | 0.366 | 0.03 | 0.445 | 0.01 |  |  | 0.262 | 0.16 | 0.349 | 0.06 |
| cg12433559 | -0.454 | 0.01 | -0.470 | 0.00 |  |  | -0.415 | 0.02 | -0.424 | 0.02 |
| cg05581469 | 0.356 | 0.04 | 0.389 | 0.02 |  |  | 0.254 | 0.18 | 0.290 | 0.12 |
| cg16994041 | 0.491 | 0.00 | 0.634 | 0.00 |  |  | 0.385 | 0.04 | 0.559 | 0.00 |
| cg22528270 | 0.472 | 0.00 | 0.565 | 0.00 |  |  | 0.392 | 0.03 | 0.500 | 0.00 |
| cg09254210 | -0.315 | 0.07 | -0.345 | 0.05 |  |  | -0.231 | 0.22 | -0.256 | 0.17 |
| cg14001486 | -0.477 | 0.00 | -0.533 | 0.00 |  |  | -0.432 | 0.02 | -0.500 | 0.00 |
| cg17306848 | -0.384 | 0.02 | -0.445 | 0.01 |  |  | -0.332 | 0.07 | -0.399 | 0.03 |
| cg00300046 | -0.351 | 0.04 | -0.427 | 0.01 |  |  | -0.296 | 0.11 | -0.382 | 0.04 |
| cg02481000 | 0.483 | 0.00 | 0.571 | 0.00 |  |  | 0.407 | 0.03 | 0.508 | 0.00 |
| cg10334053 | -0.413 | 0.02 | -0.464 | 0.01 |  |  | -0.307 | 0.10 | -0.357 | 0.05 |
| cg02707854 | 0.210 | 0.23 | 0.295 | 0.09 |  |  | 0.211 | 0.26 | 0.307 | 0.10 |
| cg03014241 | -0.415 | 0.01 | -0.433 | 0.01 |  |  | -0.350 | 0.06 | -0.366 | 0.05 |
| cg00676801 | -0.251 | 0.15 | -0.276 | 0.11 |  |  | -0.099 | 0.60 | -0.122 | 0.52 |
| cg23752651 | -0.238 | 0.18 | -0.238 | 0.17 |  |  | -0.201 | 0.29 | -0.189 | 0.32 |
| cg05599723 | 0.469 | 0.01 | 0.527 | 0.00 |  |  | 0.392 | 0.03 | 0.450 | 0.01 |
| cg15526535 | -0.353 | 0.04 | -0.383 | 0.03 |  |  | -0.260 | 0.17 | -0.290 | 0.12 |
| cg22677556 | -0.391 | 0.02 | -0.389 | 0.02 |  |  | -0.370 | 0.04 | -0.374 | 0.04 |
